# Supplementary material for: The archaeological potential of the northern Luangwa Valley, Zambia: The Luwumbu basin
Source: PLoS One. 2023 Mar 14;18(3):e0269209. doi: 10.1371/journal.pone.0269209 (PMC10013907; doi:10.1371/journal.pone.0269209)
Supplement: S1 File — Topographic and geological attributes of the survey localities. (DOCX) [file pone.0269209.s001.docx]

SI 1

| **Locality** | **Lithics** | **Elevation** | **Erosivity** | **Slope (deg.)** | **Geological Unit** | **Landcover 2016** |
| --- | --- | --- | --- | --- | --- | --- |
| SW5 | 1 | 770 | 4950.84 | 4.41 | S2 | Grassland |
| SW22 | 1 | 865 | 4984.41 | 4.07 | T | Grassland |
| SW23 | 1 | 864 | 4984.41 | 6.32 | T | Cropland |
| SW25 | 1 | 817 | 5012.95 | 3.75 | T | Shrubland |
| SW37 | 1 | 843 | 4969.96 | 4.00 | T | Shrubland |
| SW60 | 1 | 846 | 4972.91 | 4.94 | T | Shrubland |
| M01 | 1 | 784 | 4909.61 | 4.21 | T | Shrubland |
| SURVEY1 | 1 | 857 | 5002.07 | 5.67 | T | Grassland |
| SW94 | 0 | 841 | 5041.62 | 2.81 | R2 | Grassland |
| SW15 | 0 | 803 | 4982.46 | 3.55 | R3 | Grassland |
| SW69 | 0 | 799 | 4991.52 | 3.72 | R3 | Grassland |
| SW81 | 0 | 834 | 5015.68 | 0.74 | R3 | Grassland |
| SW82 | 0 | 827 | 5031.37 | 3.63 | R3 | Grassland |
| SW83 | 0 | 826 | 5030.56 | 0.00 | R3 | Cropland |
| SW86 | 0 | 827 | 5050.65 | 1.68 | R3 | Tree cover |
| SW87 | 0 | 831 | 5050.65 | 0.74 | R3 | Cropland |
| SW88 | 0 | 822 | 5050.65 | 3.63 | R3 | Cropland |
| SW89 | 0 | 829 | 5050.65 | 3.31 | R3 | Cropland |
| SW90 | 0 | 833 | 5042.05 | 3.63 | R3 | Grassland |
| SW91 | 0 | 829 | 5042.05 | 1.77 | R3 | Grassland |
| SW92 | 0 | 832 | 5042.05 | 0.74 | R3 | Grassland |
| SW93 | 0 | 838 | 5042.05 | 1.77 | R3 | Tree cover |
| SW95 | 0 | 837 | 5059.64 | 3.12 | R3 | Shrubland |
| SW66 | 0 | 813 | 4998.53 | 3.85 | S2 | Grassland |
| SW67 | 0 | 811 | 5002.90 | 3.98 | S2 | Grassland |
| SW68 | 0 | 805 | 5002.90 | 2.95 | S2 | Grassland |
| SW70 | 0 | 808 | 4989.63 | 3.50 | S2 | Tree cover |
| SW71 | 0 | 810 | 5002.90 | 3.39 | S2 | Grassland |
| SW72 | 0 | 814 | 5011.56 | 2.33 | S2 | Tree cover |
| SW73 | 0 | 814 | 5011.56 | 3.44 | S2 | Tree cover |
| SW74 | 0 | 815 | 5011.56 | 1.47 | S2 | Grassland |
| SW75 | 0 | 817 | 5011.56 | 5.13 | S2 | Grassland |
| SW76 | 0 | 820 | 5011.56 | 0.93 | S2 | Shrubland |
| SW77 | 0 | 836 | 5012.68 | 2.66 | S2 | Grassland |
| SW78 | 0 | 840 | 5012.68 | 2.11 | S2 | Tree cover |
| SW79 | 0 | 838 | 5012.68 | 3.29 | S2 | Grassland |
| SW80 | 0 | 830 | 5034.41 | 2.51 | S2 | Tree cover |
| SW96 | 0 | 830 | 5027.10 | 2.00 | S2 | Cropland |
| SW97 | 0 | 835 | 5027.10 | 6.05 | S2 | Cropland |
| SW98 | 0 | 842 | 5040.36 | 2.81 | S2 | Grassland |
| SW103 | 0 | 838 | 5039.50 | 2.33 | S2 | Cropland |
| SW104 | 0 | 840 | 5034.45 | 2.08 | S2 | Grassland |
| SW105 | 0 | 838 | 5034.41 | 6.13 | S2 | Grassland |
| SW113 | 0 | 816 | 5002.90 | 5.30 | S2 | Grassland |
| SW9 | 0 | 783 | 4955.60 | 4.65 | S3 | Grassland |
| SW16 | 0 | 804 | 4982.46 | 4.02 | T | Grassland |
| SW19 | 0 | 804 | 4975.70 | 2.31 | T | Shrubland |
| SW20 | 0 | 810 | 4995.39 | 1.86 | T | Tree cover |
| SW24 | 0 | 872 | 5004.19 | 4.93 | T | Grassland |
| SW26 | 0 | 824 | 5022.57 | 5.99 | T | Tree cover |
| SW35 | 0 | 842 | 4969.96 | 1.36 | T | Shrubland |
| SW36 | 0 | 840 | 4969.96 | 4.65 | T | Shrubland |
| SW38 | 0 | 838 | 4950.07 | 0.66 | T | Tree cover |
| SW39 | 0 | 799 | 4947.48 | 0.74 | T | Grassland |
| SW40 | 0 | 770 | 4957.37 | 2.98 | T | Grassland |
| SW56 | 0 | 804 | 4961.05 | 4.02 | T | Grassland |
| SW57 | 0 | 802 | 4961.05 | 3.31 | T | Shrubland |
| SW58 | 0 | 816 | 4966.14 | 1.65 | T | Grassland |
| SW59 | 0 | 834 | 4966.14 | 8.59 | T | Shrubland |
| SW61 | 0 | 863 | 5002.39 | 2.83 | T | Grassland |
| SW62 | 0 | 832 | 5022.57 | 1.77 | T | Tree cover |
| SM63 | 0 | 830 | 5022.57 | 1.04 | T | Shrubland |
