# Supplementary material for: The archaeological potential of the northern Luangwa Valley, Zambia: The Luwumbu basin
Source: PLoS One. 2023 Mar 14;18(3):e0269209. doi: 10.1371/journal.pone.0269209 (PMC10013907; doi:10.1371/journal.pone.0269209)
Supplement: S2 File — (DOCX) [file pone.0269209.s002.docx]

**SI 2. Supplementary Information: Micromorphology**

By Flora C. Schilt, ICArEHB, Universidade do Algarve

**Methods**

Oriented blocks of intact sediment were studied in thin section using stereo microscopes and petrographic microscopes under plane polarized light (PPL), crossed polarized light (XPL), and incident light at magnifications ranging from 20x to 500x.

Thin sections were produced from block samples collected from excavation profiles and a bedrock outcrop (Goldberg and Macphail 2003). In the lab, the samples are oven-dried for several days at 60°C and impregnated with resin under vacuum. The resin is prepared with 7 volume units of unpromoted polyester resin (Viscovoss N 55S), 3 volume units of styrene (styrene for synthesis) and 5-6ml/l hardener (methylethylketone peroxide, MEKP). After hardening, the blocks were sliced with a machine rock saw into slabs and thin section-sized chips. Selected chips were processed into c. 30-μm thick 5 x 7.5-cm sized thin sections by Spectrum Petrographics in Vancouver, WA, USA.

Micromorphological descriptions follow the work by Courty et al. (1989) and guidelines for thin section analysis by Stoops (2010). A Nikon SMZ25 stereomicroscope and Nikon Eclipse LV100D petrographic microscope with Nikon DS-Ri2 camera was used at the laboratory of the Interdisciplinary Center for Archaeology and Evolution of Human Behavior, ICArEHB, University of the Algarve, Faro, Portugal.

**Table SI 2-1.** List of micromorphological samples

| Sample | Trench, wall | Unit | # Thin sections | Comments |
| --- | --- | --- | --- | --- |
| ZA-19-01 | T2 B E-wall (ext) | 2 | 1 | Underneath cobble layer exposed on platform |
| ZA-19-02 | T2 E-wall | 4 | 2 | Very top to investigate formation of terrace and artefact context |
| ZA-19-03 | T1 S-wall | 3-4 | 4 | Top of cobbles and above: lower of U4 |
| ZA-19-04 | T1 W-wall | 4 | 2 | Between PP5 and PP6. Upper part of U4 |
| ZA-19-05 | T1 W-wall | 4 | 2 | Top of U4 and topsoil |
| ZA-19-06 |  | 1 | 1 | Dark-red sandstone (bedrock) |

**Results**


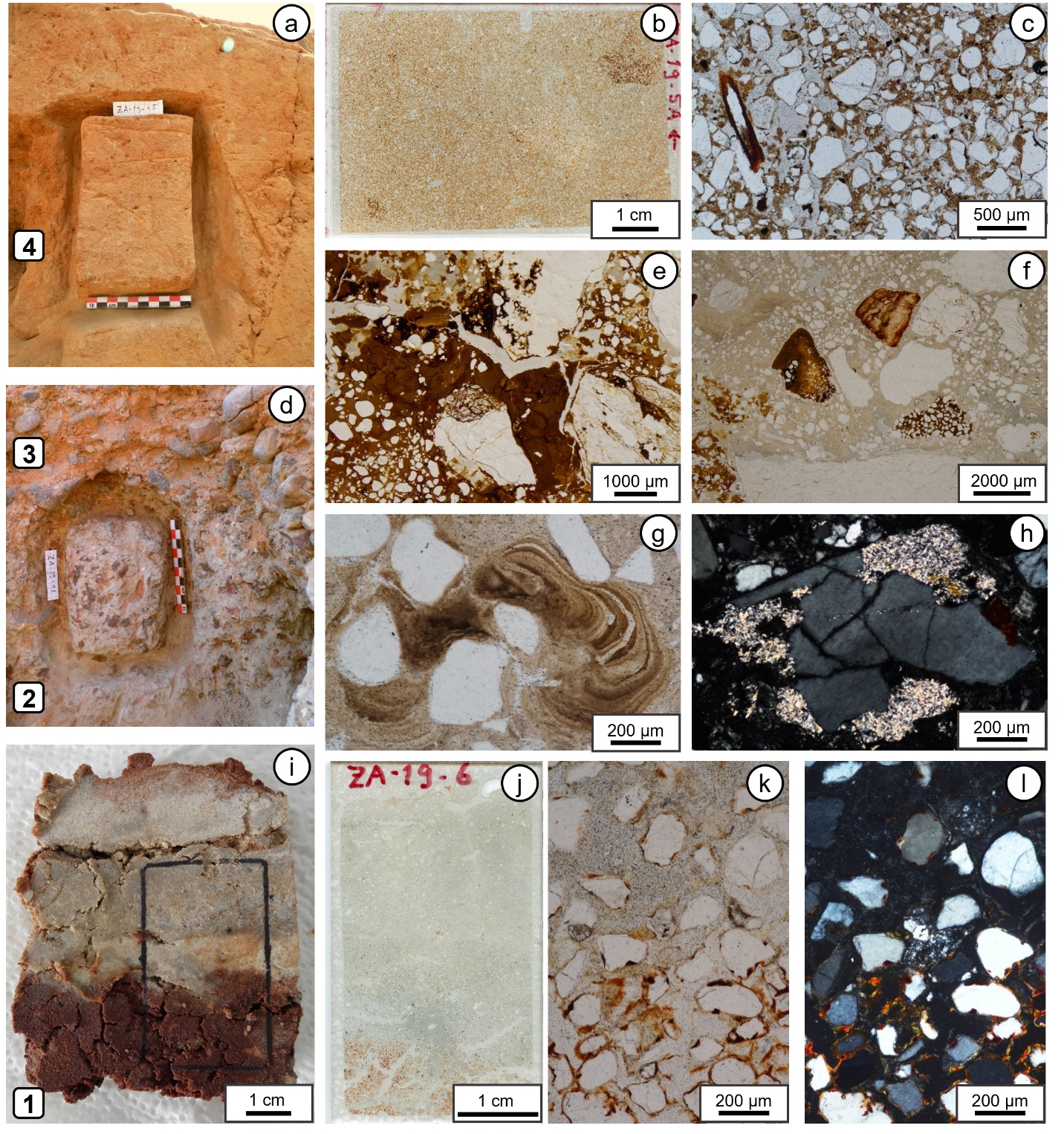


**Figure SI 2-1.** Micromorphological samples from bedrock (Unit 1) and the sedimentary sequence (Units 2-4, note square labels on the left side), illustrating characteristics discussed in the main text. (a) Sample (ZA-19-05) near the eroding surface of unit 4 before collection in the test trench (T1), displaying a homogeneous pinkish color due to lateritic soil formation and disturbances from small roots and insects. (b) Thin section scan from the sample shown in (a), in which redox masses (mottles) are visible in the top right and lower left. (c) Subangular to well-rounded fine sand in unit 4, consisting mostly of quartz with very few feldspars. The roundness of the sand reflects the relatively high maturity of the sediment, as a function of weathering from sediment transport and chemical soil weathering. Biological activity has created an open crumb microstructure (photomicrograph taken with plane polarized light [PPL]. (d) A sample (ZA-19-01) taken from unit 2, with cobbles (unit 3) visible just above. The sediment is pale (grayish) in color with distinct redox masses and reworked sandstone fragments. (e) Photomicrograph showing strong redox in the micromass (clay) between the pebbles and cobbles of Unit 3, having a cementing effect (taken with PPL and added oblique incident light). (f) Pebbles of sandstone (lower right), quartzite, feldspar (appearing white), and iron-rich chalcedony (dark-red fragments in the middle of the photomicrograph) in clay-rich sediment directly overlying the cobbles (PPL). (g) Laminated clay coatings in a black speckled micromass in unit 2. Clay illuviation and redox features near the cobble horizon likely formed in relation to groundwater movements. (h) A coarse sand grain of feldspar shows signs of weathering, observed in the fragmentation of the sand grain and sericite formation along its edges (taken with crossed polarizers). (i) Fragment of a sliced sample from sandstone bedrock of the Upper Grit Formation (ZA-19-06). While all exposed surfaces are dark red in color, deeper parts are pale and rich in clay, possibly kaolinite. The rectangular marking indicates the outline of the thin section shown in (j). (j) Thin section (c. 25 x 45 mm) of the weathering sandstone sample shown in (i). (k-l) The sand grains within the older sandstone bedrock are less rounded than in the overlying, younger, sediments, indicating that they underwent less weathering and maintained a lower maturity (compare with [c]). The outer, red, part of the rock in the lower half shows ferruginous clay coatings around the sand grains (PPL and XPL).

**Table SI 2-1**. Summary of field observations and micromorphological descriptions (next page)

|  |  |  |  | ***micromorphology*** | | | | | | | |  |
| --- | --- | --- | --- | --- | --- | --- | --- | --- | --- | --- | --- | --- |
| **Unit** | **Short field description, texture** | **Thin sections** | **Trench** | **Macro observations** | **Micro-structure &**  **c/f related distribution** | **Coarse fraction*** | **weatherable minerals** | **Fine fraction; b-fabric** | **Inclusions** | **Pedo-features** | **Interpretation** | **Arch. relevance/implications** |
| 4 (topsoil) | Sandy clay, topsoil. Many (insect and root) disturbances | 5A | T1 | Homogeneously reddened, few cm-sized dark red irregular | Close spaced fine enaulic - microaggregate microstructure | F. to c. sand | Few feldspar (f.-m. sand) | Dotted and speckled yellowish clay with red hematite; undifferentiated b-fabric | Very few v. c. sand <5%  Dispersed black (Mn) silt | Laminated clay coatings only inside redox mass (upper right) | Topsoil (until recently more deeply buried) | Easy loss (erosion) of potential (dispersed) artefact contexts |
| 4 (upper) | Sandy clay with 5-10% inclusions of subrounded to rounded coarse to very coarse sand. Many (insect and root) disturbances | 5B | T1 | Pale top pinkish red in top, common channels, roots, few dark red fragments | Close spaced fine enaulic, locally close porphyric. Mostly micro-aggregated | F. to c. sand | Few feldspars (f.-m. sand) | Speckled and dotted gray and yellowish clay; undifferentiated b-fabric | Very few v. c. sand <5% | Few clay coatings inside redox masses. Common roots and channels. | Water-lain.  Lateritic soil formation (immature, as until recently more deeply buried) | Artefacts likely in situ but moderate termite activity observed. Compaction and bioturbation may have led to slight (cm-scale) downward movement of artefacts |
| 4  (start of bioactive zone) | Sandy clay, common inclusions of very coarse sand. Common (insect and root) disturbances | 4A, 4B | T1 | Similar to the top of 3 at first sight, slightly more yellowish. cm-sized fragments of red sandstone. Faint orange redox masses | Close to single fine enaulic (aggregates); close to single porphyric (intact) | M. to c. sand with common v. c. sand | Few and mostly in finer grains (rarely coarse sand) | Pale yellowish dusty clay. Very rare weak grano-striated b-fabric (more in mottles) | Esp. A contains a few fine gravel grains | Few to common dusty clay coatings, esp. in redox masses.  Red fine sandy termite construction features. Common biochannels (roots and soil fauna) | Water-lain.  Incipient lateritic soil | Artefacts likely in situ |
| 4  Underneath terrace with concentrated artefacts | Sandy clay, laminated in the top. Very few inclusions of very coarse sand. Bioturbation disturbances | 2A, 2B | T2 | Light reddish color. Few cm-scale disintegrating dark red mottles or sandstone.  B: fresh roots, other vegetal remains, and zones with pellets (ellipsoids) soil fauna.  In A: less vegetal (root) remains, fewer bioturbation features | Close to single porphyric  (esp. close where more bioturbated)  Locally microaggregate in top few cm of A.  2B locally double spaced | V. f. to m sand | Few to common, some sericite formation | Pale yellowish speckled and dotted (Fe-oxides); undifferentiated b-fabric | Less than 5% (very) coarse sand | Few yellow dusty cc; common small orthic Fe-Mn nodules, redox masses with Fe- cc; Fresh roots and plant remains with prismatic calcium oxalate crystals; common pellets. Void spaces by bioactivity | The moderate extent of redox and roots/bioturbation indicates that this level has been near the erosional surface since some time (estimated 50 years or more) | Halting point of erosion, forming a structural terrace with artefacts and other coarse materials concentrated on top as a result of winnowing, most likely by sheetwash. |
| 4  (lower) | Sandy clay with up to 5% inclusions of very coarse sand. Common feldspars in lower. Distinct red (sandstone) mottles | 3A, 3B, 3C, 3D | T1 | Many reworked red and orange sandstone fragments following inclination of U3, and in situ redox masses | Close (3A) to double spaced (3C, 3D) porphyric | V. f. to m sand | Very few to common (fine sand). | PPL: gray to pale yellowish, very finely speckled; XPL: mostly extinct but where slightly translocated: gray to minimally yellowish. Gray areas with wrinkled fibrous authigenic clay (3C, 3D) | < than 5% (very) coarse sand | Localized cc; common discrete ca. 1 cm nodules of rounded red sandstone and broken up masses of 1.3 cm, following inclination of U3 | Water-lain | Possible in situ artefacts on top of cobble streambed |
| 3 | Imbricated cobbles and pebbles, slightly cemented | 3C, 3D | T1 | Large pebbles and cobbles, inclination of ca. 45°. Many red sandstone and other ferruginous pebbles; poss. flake lying flat (at 45° angle) on top of unit | - | Gneiss, quartzite, sandstone, some (Fe-stained) chert, chalcedony, (weathered) feldspars, rounded red sandstone | Common (but heavily weathered) | Gray colorless dusty to yellowish speckled clay | Poorly sorted sand | Distinct redox masses, many cc; large translocations of clay and silt in between cobbles, accompanied by redox | Cobble streambed of a braided (shifting) river system, or a meandering river. Post-depositional water stagnation related to fluctuations in water table and/or permeability change at the level of the cobbles (U3).  Fe oxides availability due to weatherable minerals | Artefacts have likely been transported and redeposited with the cobbles. |
| 2 | Sandy clay*. Sandstone fragments/distinct red mottles | 1 | T2 B ex | Very pale yellowish white sediment with irregular orange and dark red material. red sorted sediment in top (termite construction) | Close to single spaced porphyric | V. f. to m sand | A bit more common than in U4; common sericite formation | Gray to pale yellowish, very finely speckled. XPL: extinct but where slightly translocated gray to yellowish | One rounded spherical coarse pebble (2.5 cm) | Thick cc´s, slightly stained (Fe); prominent irregular red fragments of reworked sandstone; fragmented red crust of sorted f. s. and si; few roots; termite pellets | Water-lain, alluvial | Similar depositional environment as U4 |
| 1 | Red sandstone from lower down in canyon | 6 | Off site | Red only on outside, pale inside. Redox post-depositional | Close-spaced in red areas to localized double- spaced in colorless parts. | Subangular to subrounded v. f. to m. sand | Common | Gray to pale yellowish, very finely speckled | < 5% coarse sand; very few v. c. sand | Roots, cc | Weathering sandstone with few roots and cc. Least mature sand compared to the overlying sedimentary units. Similar depositional environment as Unit 4. | Sandstone similar to fragments redeposited in U2-4 |

* Unless indicated differently, the coarse fraction is always poorly sorted with an angularity of subangular to well-rounded. Silt is a common component in all sedimentary units.

**Abbreviations**

cc – clay coating

Si – silt

v. f. s. – very fine sand etc.

PPL – plane polarized light

XPL – crossed polarized light

**References**

Beckmann, T. (1997). Präparation bodenkundlicher Dünnschliffe für mikromorphologische Untersuchungen. In K. Stahr (Ed.), Mikromorphologische Methoden in der Bodenkunde. Hohenheimer Bodenkdl. Hefte (Vol. 40, pp. 89–103).

Bullock, P., N. Fedoroff, A. Jongerius, G. Stoops & T. Tursina (1985). Handbook for Soil Thin Section Description. Waine Research Publications, Albrighton.

Courty, M. A., P. Goldberg & R. Macphail (1989). Soils and Micromorphology in Archaeology. Cambridge: Cambridge University Press.

Goldberg, P. & R. I. Macphail (2003). Short Contribution: Strategies and Techniques in Collecting Micromorphology Samples. Geoarchaeology, 18, 571-578.

Stoops, G. (2003). Guidelines for analysis and description of soil and regolith thin sections. Madison, Wisconsin, USA: Soil Science Society of America, Inc.
