# Supplementary material for: The archaeological potential of the northern Luangwa Valley, Zambia: The Luwumbu basin
Source: PLoS One. 2023 Mar 14;18(3):e0269209. doi: 10.1371/journal.pone.0269209 (PMC10013907; doi:10.1371/journal.pone.0269209)
Supplement: S3 File — (DOCX) [file pone.0269209.s003.docx]

**Supplementary Information: Grain-size and Geochemical results**

By: Matthew Peros, Bishop’s University

**Methods**

Grain size analysis was undertaken on the <2mm fraction from each sample. Frist, each bag was mixed (homogenized) to ensure representative sampling of sediment size. Then, roughly 1 cc of sediment was collected from each sample, passed through a 2 mm sieve, and placed in a 15 ml centrifuge tube. Sodium hexametaphosphate (10%) was then added to each tube and the sample was capped and shaken by hand and left overnight. The next day, the sample was further shaken for 1 minute using a vortex mixer, and then sonicated for 1 minute to further disaggregate the finer particles. The sample was then measured using a Betatek Laser Particle Size Analyzer at the Climate and Environmental Change Research Laboratory at Bishop’s University in Sherbrooke, Quebec, Canada.

Bulk elemental geochemical analysis was undertaken on each sample using an Olympus Vanta^TM^ handheld XRF analyzer to obtain concentrations of a suite of elements, including Al, Si, P, Cl, Ca, Ti, Mn, Fe, Ni, Cu, Zn, Rb, Sr, Y, Zr, Nb, Pb, and Th.

**Results**

The grain size distributions for each sample show multi-modal distributions (Figure 1). In most cases the maximum grain size is 500-600 microns diameter (medium- to coarse-sand), whereas the minimum size is ~0.2 microns (clay)(Wentworth, 1922). Most of the distributions show modes at approximately 0.4 microns, 60-70 microns (silt-sand boundary), and 200 microns. Sample PP5 is anomalous in that it contains larger grain sizes in the coarse sand and pebble fractions. Overall, mean grain sizes (black line in Figure 1) center around 100 microns in diameter, although are slightly larger in samples PP4, PP5, and PP6.

The elemental results show generally uniform values with depth. Si, Al, Ti, and Fe are especially abundant, with values of Fe peaking toward the bottom of the section in sample PP3. The Al/Si ratio, which may indicate the presence of certain clay minerals and hence enhanced chemical weathering, is high in samples PP2 and PP3, along with Cl. Al/Si has been used as a proxy for the relative proportion of clays compared to quartz sand (R.G. & Croudace, 2015; Van Hoang et al., 2010), although a higher proportion of clays is not apparent in the grain-size results. Otherwise, there are no apparent patterns between the grain size and elemental data to report.


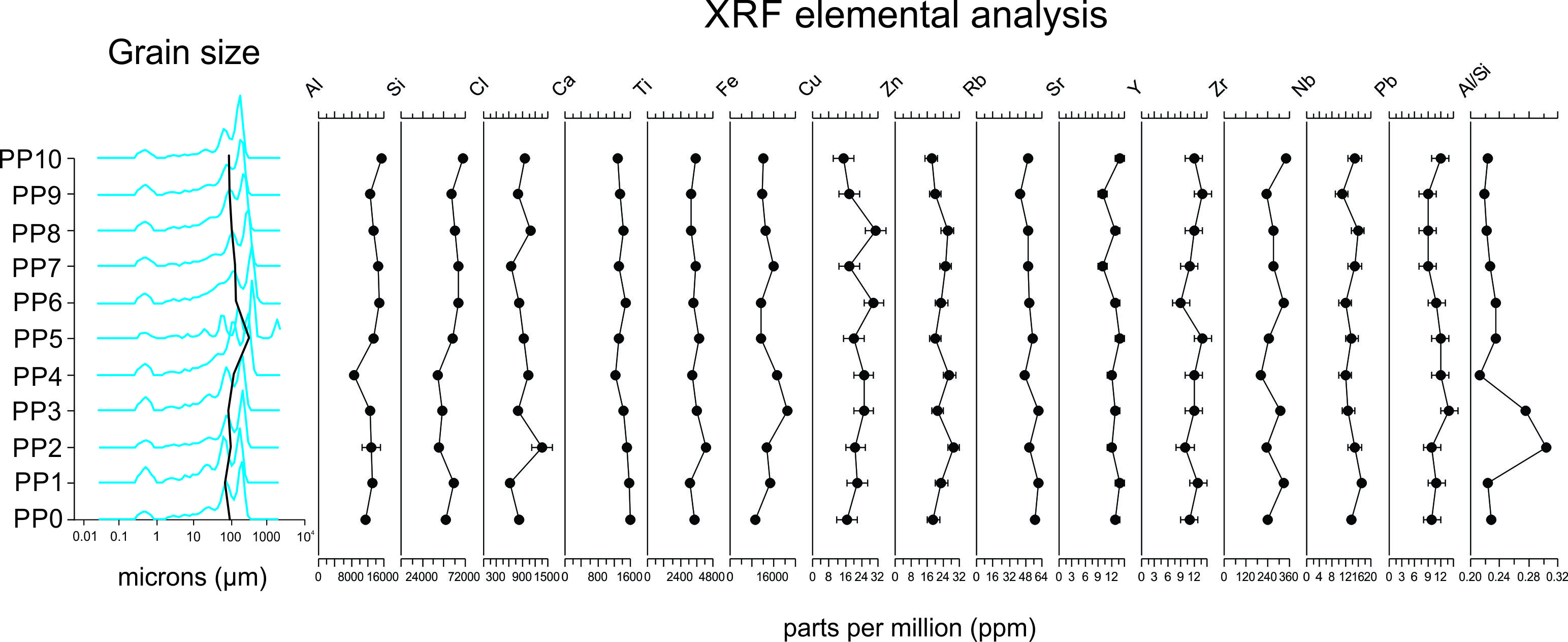


**Figure 1.** Grain size distributions (log-scale) in microns plotted in blue. The mean (in microns) of each sample is plotted using the black line. XRF elemental results for all elements that have values above detection limits are show on the right in ppm. Error bars are also plotted for each sample; often these errors are less than the size of the black circle, especially for more abundant elements (e.g., Al, Si).

**References**

R.G., R., & Croudace, I. W. (2015). Twenty years of XRF core scanning marine sediments: What do geochemical proxies tell us. In C. IW & R. RG (Eds.), *Micro-XRF Studies of Sediment Cores* (Vol. 17, pp. 25–102). Springer. <https://doi.org/10.1007/978-94-017-9849-5_2>.

Van Hoang, L., Clift, P. D., Schwab, A. M., Huuse, M., Nguyen, D. A., & Zhen, S. (2010). Large-scale erosional response of SE Asia to monsoon evolution reconstructed from sedimentary records of the Song Hong-Yinggehai and Qiongdongnan basins, South China Sea. In P. D. Clift, R. Tada, & H. Zheng (Eds.), *Monsoon Evolution and Tectonic–Climate Linkage in Asia* (Vol. 342, p. 0). Geological Society of London. <https://doi.org/10.1144/SP342.13>

Wentworth, C. K. (1922). A Scale of Grade and Class Terms for Clastic Sediments. *The Journal of Geology*, *30*(5), 377–392. <http://www.jstor.org/stable/30063207>
