# Supplementary material for: The archaeological potential of the northern Luangwa Valley, Zambia: The Luwumbu basin
Source: PLoS One. 2023 Mar 14;18(3):e0269209. doi: 10.1371/journal.pone.0269209 (PMC10013907; doi:10.1371/journal.pone.0269209)
Supplement: S4 File — (DOCX) [file pone.0269209.s004.docx]

**Supplementary Information 3 – Phytoliths**

**Extraction protocol**

Phytolith analyses were conducted on 1.5 to 3 g of sediment for each sample. The extraction protocol is based on Aleman et al. (2013), and consists of several chemical treatments : (1) deflocculation of sediments using sodium hexametaphosphate (NaPO_3_), (2) dissolution of carbonates using hydrochloric acid (HCl at 1N), (3) oxidation of the organic matter using a mix of potassium chlorate and nitric acid (Strömberg, 2004; Strömberg et al., 2018), (4) reduction and removal of iron oxides using trisodium citrate and dithionite, (5) removal of clay by gravity, (6) drying of the residue with ethanol, and (7) densimetric separation of phytoliths in a dense solution of polytungstate with a density > 2.35. A solution of Eucalyptus pollen was then added to the phytolith solution to determine phytolith influx. The recovered fraction was mounted on microscope slides using glycerin for 3D observation, and phytoliths were counted at 500X magnification. Only phytoliths with diameters larger than 5 μm were counted and classified.

**Phytolith identification**

Phytoliths were described according to their three dimensional shape and classified following the international code of phytolith nomenclature (Madella et al., 2005; Neumann et al., 2019) and based on the classification schemes of Twiss et al. (Twiss et al., 1969) and Twiss (Twiss, 1992), augmented by information presented by various studies in Africa and elsewhere (Albert et al., 2006; Aleman et al., 2014; Alexandre et al., 1997; Barboni & Bremond, 2009; Bremond et al., 2008; Fredlund & Tieszen, 1994; Garnier et al., 2013; Mercader et al., 2000, 2009; Mulholland, 1989; Neumann et al., 2009; Novello et al., 2012; Piperno, 2006; Runge, 1999). We identified and classified morphotypes and counted all phytoliths into four categories:

(1) phytoliths produced by **grasses**. Grass silica short cells (GSSC) are produced by Poaceae. Among GSSCs, we identified bilobates and crosses morphotypes, which are mainly produced by the Panicoideae subfamily and are C_4_ grasses adapted to warm and humid climate (Fredlund & Tieszen, 1994; Twiss et al., 1969). We also identified saddles, occurring principally in the Chloridoideae subfamily, and are C_4_ grasses adapted to warm and dry climate (Fredlund & Tieszen, 1994; Twiss et al., 1969). We also identified a rondel type (conical unilobate (Aleman et al., 2014)), and a trapeziform type, morphotypes mainly produced by the Pooideae subfamily (Fredlund & Tieszen, 1994; Twiss et al., 1969). Acute and fusiform-shaped types probably originate from micro-hair and prickles of grass epidermis (Bremond et al., 2005b). The flabellate types are produced in bulliform cells of the grass epidermis (Kondo et al., 1994; Twiss et al., 1969).

(2) the **woody dicotyledon category** is composed of globular granulate (Alexandre et al., 1997; Bremond et al., 2005a), globular decorated (Aleman et al., 2014; Neumann et al., 2009; Runge, 1999), blocky faceted (Aleman et al., 2014; Mercader et al., 2009; Neumann et al., 2009; Runge, 1999) and blocky granulate (Aleman et al., 2014; Mercader et al., 2009) morphotypes;

(3) two **family-specific morphotypes** were also identified: one papillae type produced by Cyperaceae in wetland (Novello et al., 2012), and the globular echinate morphotype produced by palms (Arecaceae) (Runge, 1999);

(4) finally several **non-diagnostic morphotypes** were also counted: elongated, tabular, globular smooth and blocky smooth types.

**Phytoliths indices**

Several indices can be computed to infer past vegetation structure and humidity/aridity conditions. First, the D/P index is an index of tree cover, and corresponds to the ratio of woody dicotyledon (D, globular granulate and decorated morphotypes) to Poaceae (P, GSSCs) phytoliths (Aleman et al., 2012, 2014; Bremond et al., 2005a), recently modified by Bremond et al. (Bremond et al., 2017) to be computed as D:(D+P).

The Iph index is the ratio of Chloridoideae type (saddle morphotypes) to the sum of the Chloridoideae and Panicoideae types (saddle, cross and bilobate morphotypes) (Bremond et al., 2005b, 2008; Novello et al., 2017). High Iph values (>20-40%) represent grasses dominated by Chloridoideae, and correspond to xerophytic short grass savannas, indicative of warm and dry climatic conditions. On the opposite, low Iph values indicate a dominance of Panicoideae, which are mesophytic grasses and representative of a warm and humid climate (or at least high available local soil moisture conditions).


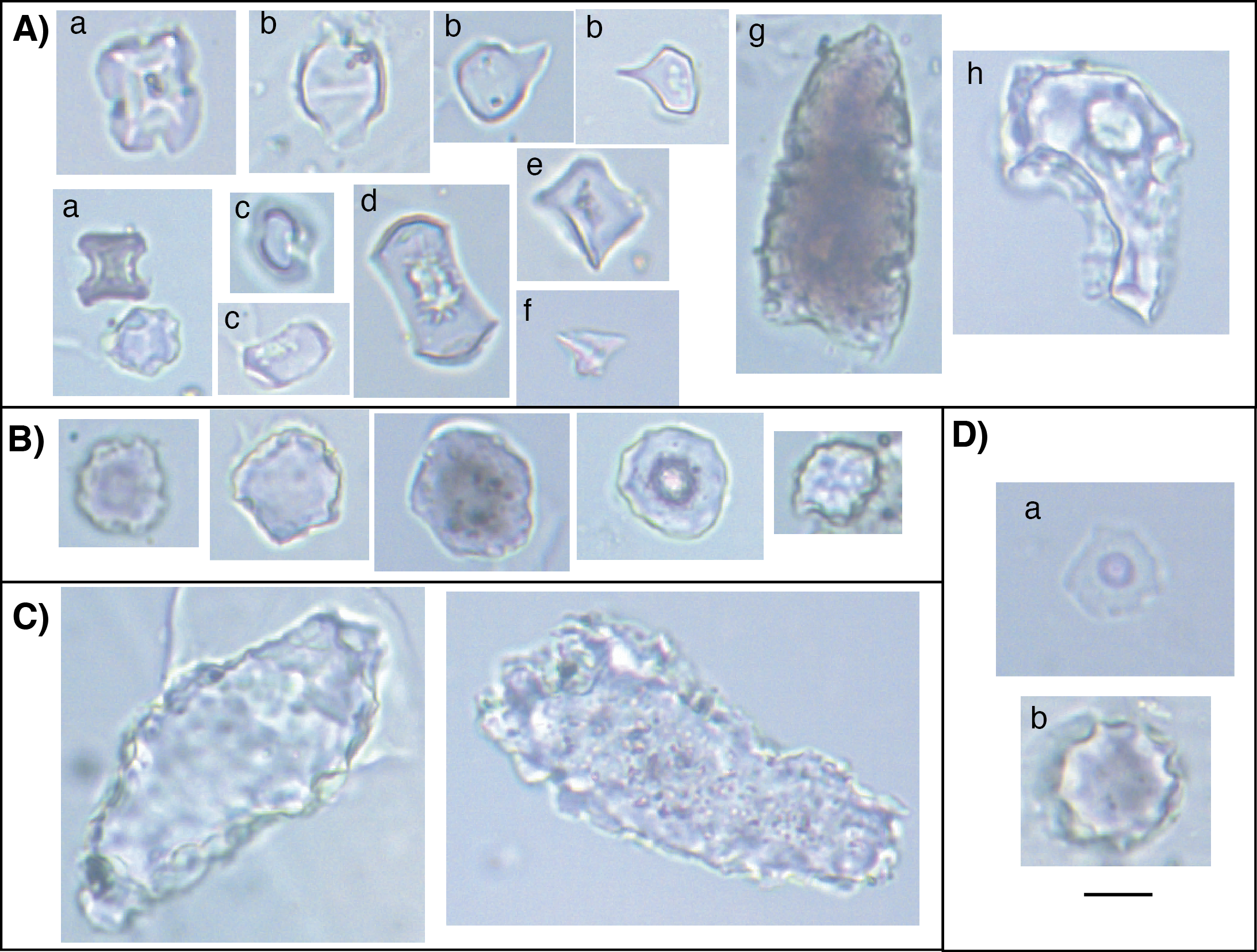


**Figure SIx-1**. Phytolith morphotypes identified in the samples and representing the four categories used in this study; scale bar = 10μm. Grass silica short cells (GSSC, A) are produced by Poaceae. Among GSSCs, crosses (a) and bilobates (b, actually half-bilobate) morphotypes, mainly produced by the Panicoideae subfamily – C_4_ grasses adapted to warm and humid climate (Fredlund & Tieszen, 1994; Twiss et al., 1969). Saddles (short (c) and long (d)) occur primarily in the Chloridoideae subfamily – C_4_ grasses adapted to warm and dry climate (Fredlund & Tieszen, 1994; Twiss et al., 1969). The rondel (f) and trapeziform (f) types are mainly produced by the Pooideae subfamily (Fredlund & Tieszen, 1994; Twiss et al., 1969). Acute and fusiform-shaped (g) types probably originate from micro-hair and prickles of grass epidermis (Bremond et al., 2005b). The flabellate types (h) are produced in bulliform cells of the grass epidermis (Kondo et al., 1994; Twiss et al., 1969). The morphotypes associated to trees (B) are principally composed of globular granulate (Alexandre et al., 1997; Bremond et al., 2005a). Other morphotypes are also associated to trees (C) like the blocky faceted (a; Aleman et al., 2014; Mercader et al., 2009; Neumann et al., 2009; Runge, 1999) and blocky granulate (d; Aleman et al., 2014; Mercader et al., 2009) morphotypes. Finally, two family-specific morphotypes (D) were also identified: one papillae type (a) produced by Cyperaceae in wetland (Novello et al., 2012), and the globular echinate (b) morphotype produced by palms (Runge, 1999).


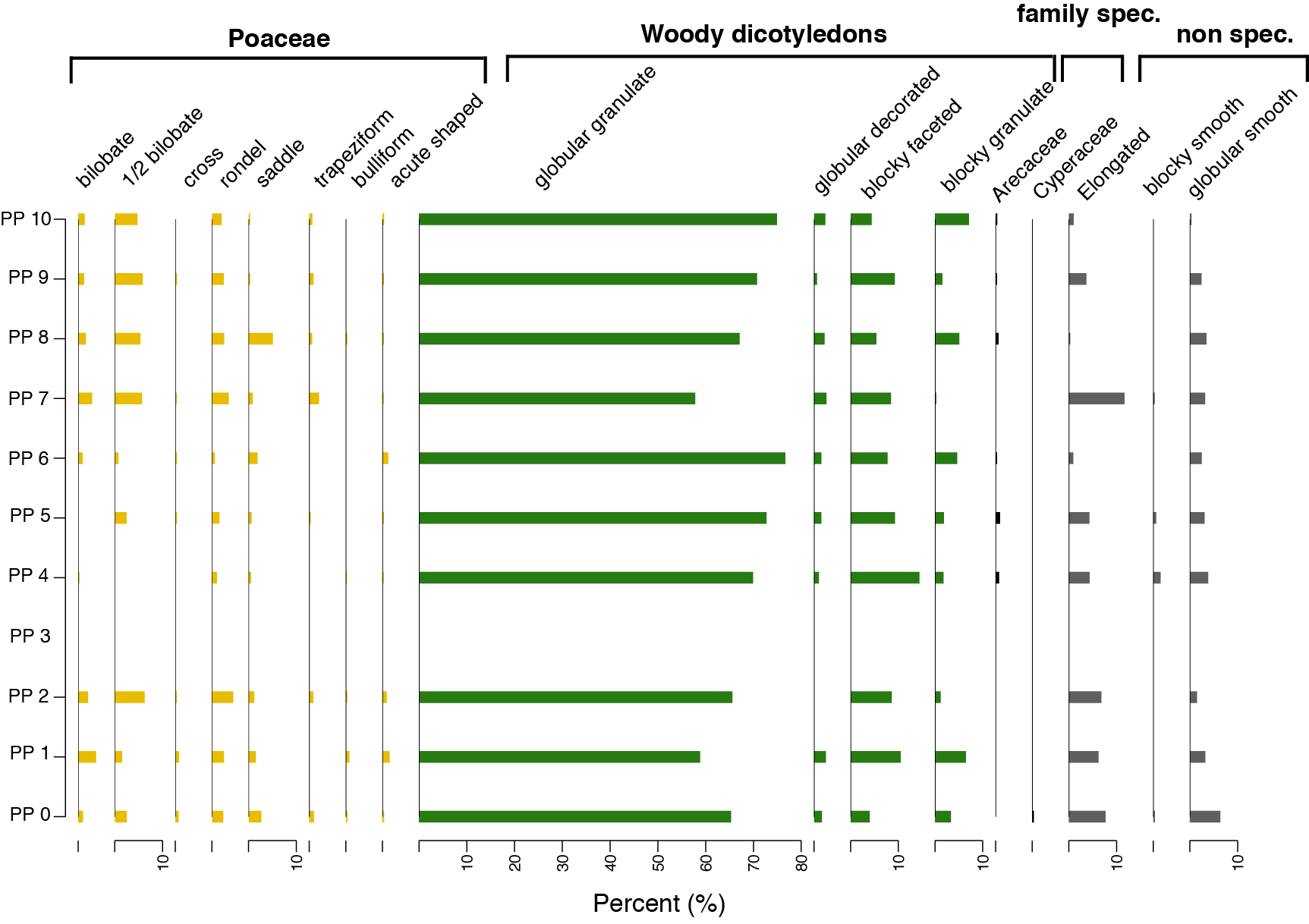


**Figure SIx-2.** Phytoliths diagrams of the total assemblage organized by group, Poaceae phytoliths (GSSC, acicular and bulliform cells), woody dicotyledons, family specific and non-specific morphotypes. The percentages were computed for the total assemblage.


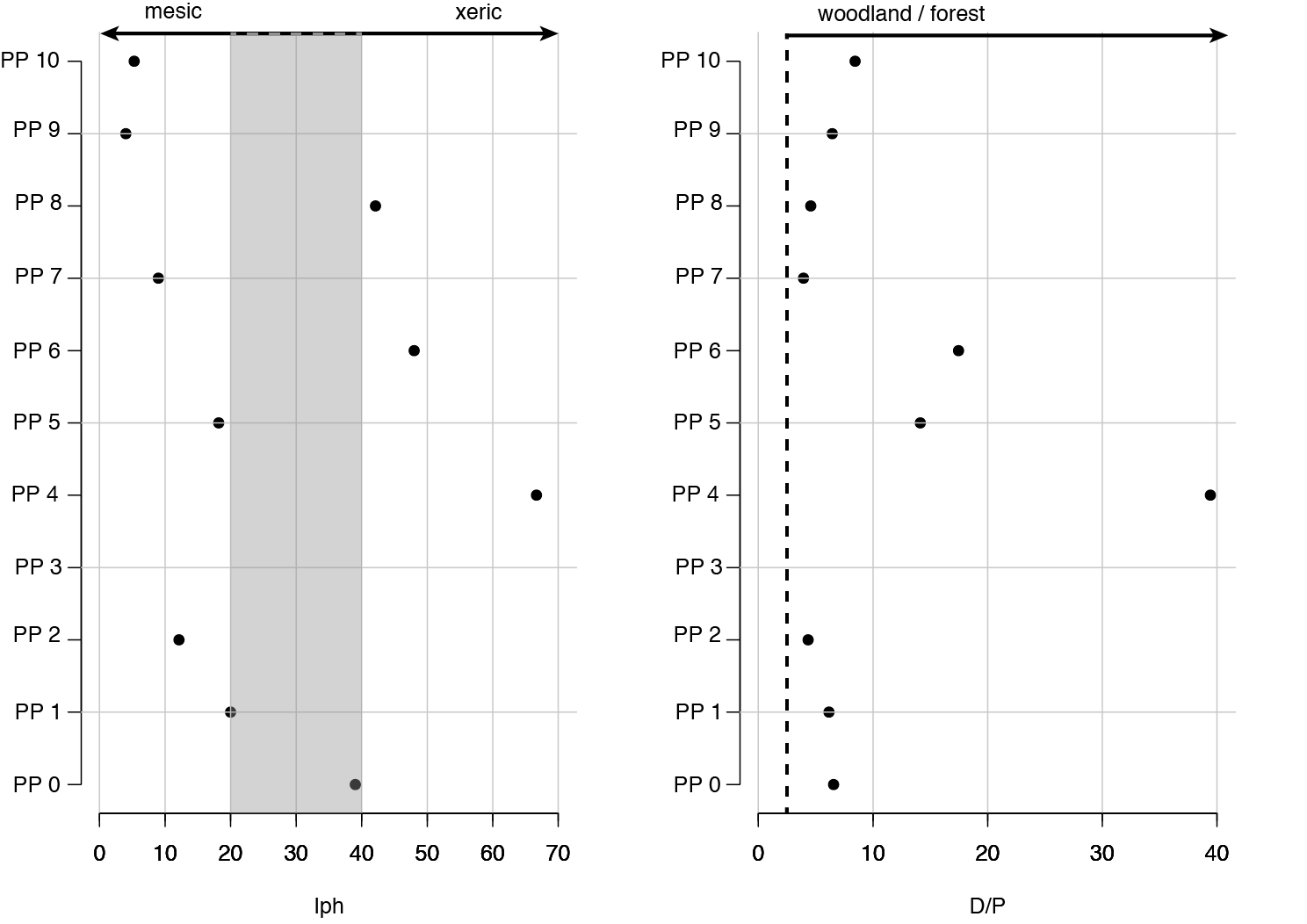


**Figure SIx-3.** Phytolith indices for the 11 samples, Iph corresponds to the aridity index and D/P to the tree cover index.

References:

Albert, R. M., Bamford, M. K., & Cabanes, D. (2006). Taphonomy of phytoliths and macroplants in different soils from Olduvai Gorge (Tanzania) and the application to Plio-Pleistocene palaeoanthropological samples. *Quaternary International*, *148*(1), 78–94.

Aleman, J. C., Leys, B., Apema, R., Bentaleb, I., Dubois, M. A., Lamba, B., Lebamba, J., Martin, C., Ngomanda, A., Truc, L., Yangakola, J.-M., Favier, C., & Bremond, L. (2012). Reconstructing savanna tree cover from pollen, phytoliths and stable carbon isotopes. *Journal of Vegetation Science*, *23*(1), 187–197.

Aleman, J. C., Saint-jean, A., Leys, B., Carcaillet, C., Favier, C., & Bremond, L. (2013). Estimating phytolith influx in lake sediments. *Quaternary Research*, *80*, 341–347.

Aleman, J. C., Canal-Subitani, S., Favier, C., & Bremond, L. (2014). Influence of the local environment on lacustrine sedimentary phytolith records. *Palaeogeography, Palaeoclimatology, Palaeoecology*, *414*, 273–283.

Alexandre, A., Meunier, J. D., Lézine, A. M., Vincens, A., & Schwartz, D. (1997). Phytoliths: indicators of grassland dynamics during the late Holocene in intertropical Africa. *Palaeogeography Palaeoclimatology Palaeoecology*, *136*(1–4), 213–229.

Barboni, D., & Bremond, L. (2009). Phytoliths of East African grasses: An assessment of their environmental and taxonomic significance based on floristic data. *Review of Palaeobotany and Palynology*, *158*(1–2), 29–41.

Bremond, L., Alexandre, A., Hely, C., & Guiot, J. (2005a). A phytolith index as a proxy of tree cover density in tropical areas: Calibration with Leaf Area Index along a forest-savanna transect in southeastern Cameroon. *Global and Planetary Change*, *45*(4), 277–293.

Bremond, L., Alexandre, A., Peyron, O., & Guiot, J. (2005b). Grass water stress estimated from phytoliths in West Africa. *Journal of Biogeography*, *32*(2), 311–327.

Bremond, L., Alexandre, A., Wooller, M. J., Hély, C., Williamson, D., Schäfer, P. A., Majule, A., & Guiot, J. (2008). Phytolith indices as proxies of grass subfamilies on East African tropical mountains. *Global and Planetary Change*, *61*(3–4), 209–224.

Bremond, L., Bodin, S. C., Bentaleb, I., Favier, C., & Canal, S. (2017). Past tree cover of the Congo Basin recovered by phytoliths and δ 13 C along soil profiles. *Quaternary International*, *434*, 91–101.

Fredlund, G. G., & Tieszen, L. T. (1994). Modern Phytolith Assemblages from the North-American Great-Plains. *Journal of Biogeography*, *21*(3), 321–335.

Garnier, A., Neumann, K., Eichhorn, B., & Lespez, L. (2013). Phytolith taphonomy in the middle-to late-Holocene fluvial sediments of Ounjougou (Mali, West Africa). *The Holocene*, *23*, 416–431.

Kondo, R., Childs, C., & Atkinson, I. (1994). *Opal phytoliths of New Zealand* (Vol. 85). Manaaki Whenua Press: Lincoln, NZ.

Madella, M., Alexandre, A., & Ball, T. (2005). International code for phytolith nomenclature 1.0. *Annals of Botany*, *96*(2), 253–260.

Mercader, J., Runge, F., Vrydaghs, L., Doutrelepont, H., Ewango, C. E. N., & Juan-Tresseras, J. (2000). Phytoliths from archaeological sites in the tropical forest of Ituri, Democratic Republic of Congo. *Quaternary Research*, *54*(1), 102–112.

Mercader, J., Bennett, T., Esselmont, C., Simpson, S., & Walde, D. (2009). Phytoliths in woody plants from the Miombo woodlands of Mozambique. *Annals of Botany*, *104*(1), 91–113.

Mulholland, S. C. (1989). Phytolith shape frequencies in North Dakota grasses: a comparison to general patterns. *Journal of Archaeological Science*, *16*(5), 489–511.

Neumann, K., Fahmy, A., Lespez, L., Ballouche, A., & Huysecom, E. (2009). The Early Holocene palaeoenvironment of Ounjougou (Mali): Phytoliths in a multiproxy context. *Palaeogeography, Palaeoclimatology, Palaeoecology*, *276*(1–4), 87–106.

Neumann, K., Strömberg, C. A. E., Ball, T., Albert, R. M., Vrydaghs, L., & Cummings, L. S. (2019). International Code for Phytolith Nomenclature (ICPN) 2.0. *Annals of Botany*, *124*(2).

Novello, A., Barboni, D., Berti-Equille, L., Mazur, J. C., Poilecot, P., & Vignaud, P. (2012). Phytolith signal of aquatic plants and soils in Chad, Central Africa. *Review of Palaeobotany and Palynology*, *178*, 43–58.

Novello, A., Barboni, D., Sylvestre, F., Lebatard, A.-E., Paillès, C., Bourlès, D. L., Likius, A., Mackaye, H. T., Vignaud, P., & Brunet, M. (2017). Phytoliths indicate significant arboreal cover at Sahelanthropus type locality TM266 in northern Chad and a decrease in later sites. *Journal of Human Evolution*, *106*, 66–83.

Piperno, D. R. (2006). *Phytoliths: a comprehensive guide for archaeologists and paleoecologists*. Altamira Pr.

Runge, F. (1999). The opal phytolith inventory of soils in central Africa--quantities, shapes, classification, and spectra. *Review of Palaeobotany and Palynology*, *107*(1–2), 23–53.

Strömberg, C. A. E. (2004). Using phytolith assemblages to reconstruct the origin and spread of grass-dominated habitats in the great plains of North America during the late Eocene to early Miocene. *Palaeogeography, Palaeoclimatology, Palaeoecology*, *207*(3), 239–275.

Strömberg, C. A. E., Dunn, R. E., Crifò, C., & Harris, E. B. (2018). Phytoliths in paleoecology: analytical considerations, current use, and future directions. In *Methods in Paleoecology* (pp. 235–287). Springer.

Twiss, P. C. (1992). Predicted world distribution of C3 and C4 grass phytoliths. In P. Press (Ed.), *Phytolith Systematic emerging issues. Advance Archaeological Museum Science*.

Twiss, P. C., Suess, E., & Smith, R. M. (1969). Morphological classification of grass phytoliths. *Soil Science Society of America Journal*, *33*(1), 109–115.
